# Parliament and Publications

**Published:** 1911-02-18

**Authors:** 


### Parliament and Publications.

### EXPERIMENTS ON DOGS.

In the House of Commons on February 13, Sir Frederick Banbury introduced a Bill to prohibit experiments upon dogs.

### RAILWAY ACCIDENTS.

The number of persons killed on railways in the United Kingdom in the course of public traffic, during the three months ended September 30, 1910, was 268, and the number of persons injured was 2,407.—"Railway Accidents," Cd. 5493, price 11½d.

### "606" AND THE ARMY.

In reply to a question asked by Mr. Goldman, the Secretary of State for War replied that steps had been taken to employ "606" in suitable cases in the British Army, and he referred the honourable member to the *Journal* of the Army Medical Corps for November, 1910, and February, 1911, for accounts of the cases in which the remedy had been tried.

### MEDICAL INSPECTION OF SCHOOL CHILDREN.

Mr. Charles Bathurst having asked a question concerning the medical inspection of school children, the President of the Board of Education referred the honourable member to the report of the Board's Chief Medical Officer for the year 1909, which was published in December last.

### SANATORIA FOR THE POOR.

Mr. Leach asked the President of the Local Government Board if he would take steps to urge all public authorities under the direction of his Department to provide sanatoria for the benefit of those people who are too poor to pay the fees at existing private establishments. Mr. Burns replied that arrangements for the treatment of the sick had already been made by boards of guardians by the provision of separate institutions or sick wards, and that in suitable cases he was urging the combination of such bodies for the provision of special institutions where these are required. Sanitary authorities have also provided about 700 hospitals, with approximately 20,000 beds, mostly for the isolation of infectious disease.

### THE PLAGUE IN MANCHURIA.

Mr. Allan Baker asked the Secretary of State for Foreign Affairs if he could say what precautions were being taken by the Chinese Government to ensure the safety of European doctors working in the plague district of Harbin. Sir Edward Grey replied as follows: "His Majesty's Minister at Pekin reports that all the doctors have been inoculated with Haffkine's vaccine. They are comfortably installed in a railway car with the exception of one, who is living with the British Consul. The Chinese Government have done their utmost for the personal comfort of the doctors, whose private letters show appreciation of their considerate treatment. They wear masks, oilskin boots, and overalls when dealing with cases of plague. The French doctor Mesny, who was not inoculated, became infected owing to a patient coughing in his face while examining him unmasked. Dr. Jackson, the British doctor, died after continuous hard work among a crowd of infected coolies. It is believed that he was too exhausted to maintain proper precautions. The heroism of the doctors, Sir John Jordan reports, is beyond praise."

### CORONERS' COMMITTEE.

A further report of the Departmental Committee appointed to inquire into the law relating to coroners and coroners' inquests, and into the practice in coroners' courts, has been issued. It contains the questions addressed to the coroners of England and Wales, together with a summary and analysis of the replies received; a memorandum handed in on behalf of the London County Council; a memorandum handed in on behalf of the British Medical Association; a return for England and Wales of cases in which deaths of persons while under the influence of anæsthetics were reported to the coroner during the year 1908; a report on the examination of the samples of flannelette referred to the Government Laboratory by the coroners' committee; memoranda on flannelette by Dr. W. H. Perkin, Mr. M. J. Riley, Mr. C. H. Whipp, and Mr. S. Bradbury; and further matter dealing with flannelette.—"Coroners' Committee" (Part III.), Cd. 5492, price 2s. 5d.

### THE HEALTH OF THE ARMY.

The improvement in the health of the troops which has been noticeable since 1903 continued in 1909. During the four years which ended December 1908, the improved health of the Army permitted the closing down of 2,200 beds at Home stations, while in the Colonies the number has been reduced by 901. The continued education of the soldier in matters of personal hygiene, further improvements in the sanitary condition of barracks and camps, and the advance of science in the direction of disease prevention will ensure the maintenance of the present satisfactory state of the health of the Army. Except as regards death-rate and average duration of each case of sickness, which were slightly higher in 1909 than in 1908, in all respects there was a decided improvement in the health and health efficiencies of the troops quartered in the United Kingdom in the year 1909, as compared with the previous year. Judging from the death-rate alone, it would appear that the health and vitality of the troops in the United Kingdom are superior to those of males of the same ages among the civil population. Turning to the Colonies the utmost satisfaction is derived from the result of a determined attack on the malarial mosquito in South China and Mauritius. At the former station the admission ratio was 138.4 per 1,000, as compared with 256.0 in 1908. In Mauritius the ratio of admissions decreased from 299.0 in 1908 to 99.0 in 1909. Taking the admission ratio for all diseases, there is a decrease compared with 1908 and the preceding five years in nearly all the commands. India shows a very marked all-round improvement. The report for the year 1909, which has only just made its belated appearance, states that during that period 50,298 men were medically examined for approval as recruits, and 15,041 were rejected as unfit for the army, a ratio of 299.04 per 1,000 as compared with a ratio of 282.21 rejections in 1908. A want of maturity has always been the main defect of recruits, and in the year under review there are no grounds for showing that this defect has been diminished to any appreciable extent. However, considering the age and physical standards required for enlistment the quality of the recruit was, on the whole, satisfactory, and with few exceptions should develop into a good stamp of soldier. The rejections for loss and decay of many teeth amount to 18 per cent. of the total rejections for all causes, and the ratio is slightly increasing. The ratio of rejections for defective vision, on the other hand, shows a satisfactory reduction.—"Report on the Health of the Army," Cd. 5477, price 1s. 10d.

